# Abubidentin A, New Oleanane-type Triterpene Ester from *Abutilon bidentatum* and its antioxidant, cholinesterase and antimicrobial activities

**DOI:** 10.7717/peerj.13040

**Published:** 2022-03-08

**Authors:** Gadah A. Al-Hamoud, Nawal M. Al-Musayeib, Musarat Amina, Sabrin R.M. Ibrahim

**Affiliations:** 1 Pharmacognosy, College of Pharmacy, King Saud University, Riyadh, Saudi Arabia; 2 Pharmacognosy, Faculty of Pharmacy, Assiut University, Assiut, Egypt

**Keywords:** *Abutilon bidentatum*, Malvaceae, Abubidentin A, Oleanane-type triterpene, Antioxidant, Cholinesterase, Antimicrobial

## Abstract

**Background:**

This work describes the phytochemical and biological investigation of aerial parts of *Abutilon bidentatum* Hochst. Of Saudi origin.

**Methodology:**

Petroleum ether fraction of ethanolic extract *A. bidentatum* was fractionated on a silica gel column and further purified with different chromatographic procedures for the isolation of chemical compounds. The chemical structures of all the pure isolated compounds were elucidated by the interpretation of their spectral data using IR, UV, ^1^H, ^13^C NMR, and MS spectroscopy and chemical methods (alkaline hydrolysis) as well as comparison with data reported in the literature. The extract and isolated compounds were evaluated for antioxidant, cholinesterase inhibitory, and antimicrobial activities.

**Results:**

A new oleanane-type triterpene ester, namely abubidentin A (**3**) (α, 3β, 30-trihydroxy-29-carboxy-olean-9(11), 12-diene-3-dotriacontanoate), along with two known compounds: 2-hydroxydocosanoic acid (**1**) and stigmasta-22-ene-3-β-ol (**2**) were isolated from the aerial parts of *Abutilon bidentatum* Hochst. (Malvaceae). Concerning the biological potential, the abubidentinA displayed antioxidant, cholinesterase inhibitory and antimicrobial activities. AbubidentinA possessed strong antioxidant activity against DPPH and ABTS^+^ radical scavenging assays. This new triterpene exhibited high inhibition against acetylcholinesterase (IC_50_ 38.13 ± 0.07 µgmL^−1^) and butyrylcholinesterase (IC_50_ 32.68 ± 0.37 µgmL^−1^). Abubidentin A displayed promising antimicrobial activity against *Escherichia coli*, *Pseudomonas aeruginosa*, and *Staphylococcus aureus* (125–150 µgmL^−1^).

**Conclusion:**

These findings suggest *A. bidentatum* can contribute as a source of new biologically active compounds, especially antioxidants and antimicrobial agents.

## Introduction

Abutilon Miller is the extensive genus of family Malvaceae that contains around 150 species of annual or perennial herbs, shrubs or undershrubs, and small trees. This plant specie is native to temperate, subtropical, and tropical areas of Africa, Asia, America and Australia (Arbat, 2012). The word Abutilon is the Greek ancient name of the mulberry tree and has been assigned to genus due to its close similarity to the morphology of leaves. The genus Abutilon showed the existence of precious insoluble fibers isolated from different species of this genus and has noteworthy importance (Gomaa et al., 2016). The species of Abutilon genus have been used to cure specific health issues including; rheumatoid arthritis, diuretic and demulcent due to the occurrence of high mucilage content (Ali et al., 2014; Baquar, 1989). Numerous important pharmacological attributes such as hepatoprotective, antioxidant, analgesic, antipyretic, anti-inflammatory, antimicrobial, anticancer, diuretic, anti-hyperglycemic, CNS activity, immunostimulant, anti-hyperlipidemias, anti-hypertensive, antidiarrheal, anti-urolithiatic, and wound healing activities have been assigned to the plant species of this genus (Khadabadi & Bhajipale, 2010; Agrawal, 2017). Phytochemical studies claimed that the genus contain different secondary metabolites including phenolic acids, flavonoids, triterpenes, sterols, coumarins, quinones, alkaloids, anthocyanins, iridoids, saponins, megastigmanes, and fatty acids (Gomaa et al., 2018). In Saudi Arabia, it is represented by five species, namely *A. bidentatum*, *A. figarianum*, *A. fruticosum*, *A. hirtum*, and *A. pannosum* (Migahid, 1978).

**Figure 1:**
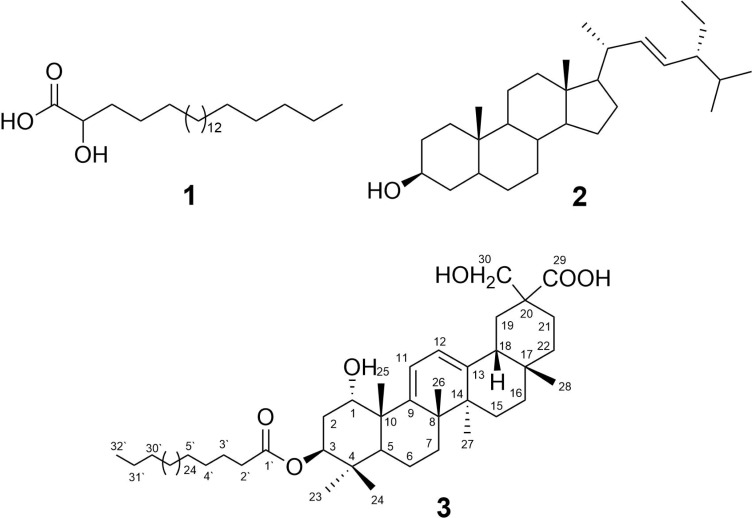
Structures of isolated compounds **1**–**3**.

*Abutilon bidentatum* Hochst. locally known as Ren-Umbro is an erect shrub, 1–2 m in height with stellate pubescent branches mixed with long simple hairs. Leaves broadly ovate, long-petiolate blade up to c. 12(−17) × 10(−13) cm, deeply cordate at base, sparse, stellate tomentose, serrate, acuminate at apex, velvety on both sides with serrate-dentate margins. Flowers are present in the leaf axils or on a shoot axillary. Seeds are papillose, 2.5 mm long stellately spreading, and black. Different parts of *A.bidentatum* have been utilized to treat several aliments in ethno-medicine, especially its leaves are used to cure infections. Root powder is used in folk-traditional medicine to treat dysentery, colitis, and diarrhea (Al-Shanwani, 1996). Literature scrutiny revealed that this plant remained unexplored for phytochemical and biological properties. Only three studies could be traced concerning the chemical constituents and pharmacological activities till date. These studies reported the antibacterial, antioxidant (Survase, Jamdhade & Chavan, 2012; Shahwar et al., 2010) and hepatoprotective potential of aerial parts of *A. bidentatum* (Yasmin, Akram Kashmiri & Anwar, 2011). However, a single study with respect to its chemical constituents, reporting the isolation of cholestane derivative from the *A. bidentatum* has been reported in the literature (Jain, Jain & Arora, 1996). The present study reports the isolation and structural characterization of a new oleanane-type triterpene ester: abubidentin A (**3**, Fig. 1) and two known compounds (**1** and **2**) from the aerial parts of *A. bidentatum*.

## Materials & Methods

### Chemicals and reagents

All the chemicals and reagents used for this study including; ethanol (96%), methanol (99.8%), petroleum ether (40–60 °C), dichloromethane (≥99.8%), chloroform (99.9%), n-butanol (99.8%), dimethyl sulfoxide (DMSO), hydrochloric acid (HCl), potassium hydroxide (KOH, ≥85%), 1,1-diphenyl2-picrylhydrazyl (DPPH, 95%), 2, 20-azino-bis[3-ethyl benzo thiazoline-6-sulphonic acid] (ABTS), potassium persulfate (K_2_S_2_O_8_), acetylthiocholine iodide (≥99.0%), S-butyrylthiochoilne iodide (≥98.0%), sodium phosphate (Na_3_PO_4_, 96%), ascorbic acid, resazurin, 2-nitrobenzoic acid and were obtained from Sigma Aldrich (Hamburg, Germany). Muller Hinton agar and Muller Hinton broth were procured from HiMedia (Himedia Laboratories Pvt Ltd., Mumbai, India). Donepezil, galantamine and chloramphenicol were purchased from the local drug store.

### Instrumentation

Optical rotation: Perkin-Elmer Model 341 LC polarimeter (Perkin-Elmer, Waltham, MA, USA). UV spectra: in MeOH using a Perkin-Elmer Lambda 25 UV/VIS spectrophotometer (Perkin-Elmer, Waltham, MA, USA). IR spectra: Shimadzu Infrared-400 spectrophotometer (Shimadzu, Kyoto, Japan). ESIMS spectra: Agilent 6320 Ion trap mass spectrometer (Agilent Technologies, Santa Clara, CA, USA) equipped with an electrospray ionization interface. NMR spectra: Bruker DRX 700 spectrometer (Bruker, Rheinstetten, Germany). Column chromatographic separations: silica gel 60 (0.04–0.063 mm, Merck, Darmstadt, Germany). TLC analyses: pre-coated silica gel F254 aluminum sheets (Merck, Darmstadt, Germany).

### Enzymes and pathogenic strains

The acetylcholinesterase (AChE) and butyrylcholinesterase (BChE) were obtained from the mouse brain and human blood at the Pharmacology Department of King Saud University, SaudiArabia. *Escherichia coli* (ATCC 25922), *Pseudomonas aeruginosa* (ATCC 27853), and *Staphylococcus aureus* were provided by Microbiology Department, King Khaled University Hospital (KKUH), Saudi Arabia.

### Plant material

The fresh aerial parts of *Abutilon bidentatum* Hochst. were collected from Jazan city, Aseer region, Saudi Arabia in March 2009. The plant was kindly identified by Dr. Mohamed Yusuf at the Pharmacognosy Department, College of Pharmacy, King Saud University. A voucher specimen (#16022) was deposited in the herbarium of the Pharmacognosy Department.

### Extraction and isolation

Air dried-powdered aerial parts of *A. bidentatum* (900 g) were exhaustively extracted by maceration with 96% EtOH (3L × 7) with shaking. The combined EtOH extract was dried under reduced pressure at 45 °C to give a dry total extract weighing 89 g (EE). A part of the dried residue (87 g) was suspended in 450 mL distilled water and fractionated by shaking with petroleum ether (500 mL × 6), dichloromethane (500 mL × 4), ethyl acetate (500 mL × 5), and n-BuOH (500 mL × 6), respectively. Each combined extract was individually evaporated under reduced pressure to give the petroleum ether (PEF, 13 g), dichloromethane (DCF, 4 g), ethyl acetate (EAF, 6 g), n-BuOH (BF, 9 g), and aqueous soluble (AF, 55 g) fractions. Therefore, part of PEF (11.5 g) was chromatographed on SiO_2_ column (330 g, 5 × 90 cm). Elution was started with petroleum ether and the polarity was gradually increased with CH2Cl2, followed by CH_2_Cl_2_: MeOH mixtures up to 100% MeOH to afford seven subfractions: *AB-1* to *AB-7*. TLC pattern of subfractions *AB-2* and *AB-5* showed the presence of prominent compounds and were selected for further column chromatography. Subfraction *AB-2* (33 mg) was chromatographed over silica gel using petroleum ether:CH_2_Cl_2_ gradient to give **1** (6.7 mg, white powder). Subfraction *AB-5* (311 mg) was applied on SiO_2_ column (15 g, 70 × 1 cm), using petroleum ether:CH_2_Cl_2_ (95:5 to 80:20) to give two subfractions *AB-5.1* and *AB-5.2* containing two major spots on TLC. Subfraction *AB-5.1* (43 mg) was purified on SiO_2_ column, eluted with petroleum ether:CH_2_Cl_2_ gradient to yield **2** (10 mg, white amorphous powder). Sub fraction *AB-5.2* was similarly treated as subfraction *AB-5.1* to give **3** (12 mg, white amorphous powder).

### Abubidentin A (1*α*,3*β*,30-Trihydroxy-29-carboxy-olean-9(11), 12-diene-3-dotriacontanoate; 3)

White amorphous powder. [*α*]_D_ +45 (*c* = 0.1, MeOH). UV (MeOH) *λ*_max_ (log *ɛ*): 212 (3.10), 275 (2.21) nm. IR (KBr): 3,420, 2,947, 1,732, 1,712, 1,636, 1,245 cm^−1^. NMR data (CDCl_3_, 700 and 176 MHz) see Table 1. (+) ESI-MS *m*/*z:* 971.4 [M+Na]^+^; (-) ESI-MS *m*/: *z* 947.8 [M-H]^-^.

**Table 1:** NMR spectral data of compound **3** (500 and 125 MHz).

Position	δ_H_ [mult., *J* (Hz)]	δ_C_ (mult.)	HMBC
1	4.17 (*d*, *J* = 2.8)	71.6 CH	2, 3, 5, 10, 25
2	1.65–1.66 (m)	30.4 CH_2_	3, 4, 10
	1.42–1.43 m		
3	5.09 (*dd*, *J* = 12.0, 5.0)	74.3 CH	1′, 1, 4, 23, 24
4	–	39.6 C	–
5	1.48–1.50 (m)	44.0 CH	3, 4, 10, 24, 25
6	1.61–1.63 (m) 1.45–1.47 (m)	16.2 CH_2_	–
7	2.05–2.07 (m) 1.81–1.82 (m)	30.9 CH_2_	–
8	–	37.1 C	–
9	–	149.5 C	–
10	–	43.4 C	–
11	5.73 (*d*, *J* = 6.0)	116.0 CH	8, 9, 10, 13
12	5.58 (*d*, *J* = 6.0)	119.6 CH	9, 14, 18
13	–	146.0 C	–
14	–	39.9 C	–
15	1.68–1.70 (m) 1.25–1.28 (m)	24.1 CH_2_	13
16	1.88–1.91 (m) 0.98–1.00 (m)	24.6 CH_2_	14
17	–	32.8 C	–
18	2.18 (*dd*, *J* = 12.8, 5.6)	41.4 CH	12, 13, 19, 22, 29, 30
19	1.63–1.65 (m) 1.05–1.08 (m)	45.1 CH_2_	17, 21, 29, 30
20	–	30.2 C	
21	1.15–1.17 (m) 1.00–1.02 (m)	33.5 CH_2_	29, 30
22	1.48–1.50 (m) 1.32–1.34 (m)	33.8 CH_2_	
23	0.97 (s)	24.6 CH_3_	3, 4, 5, 24
24	0.91 (s)	15.8 CH_3_	3, 4, 5, 23
25	1.26 (s)	22.5 CH_3_	1, 5, 9
26	1.17 (s)	19.4 CH_3_	9, 14
27	1.07 (s)	19.2 CH_3_	8, 13
28	0.92 (s)	27.2 CH_3_	17, 18, 22
29	–	173.0 C	–
30	4.06 (*d*, *J* = 10.2) 3.81 (*d*, *J* = 10.2)	69.6 CH_2_	19, 20, 21, 29
1′	–	172.3 C	–
2′	2.33 (*t*, *J* = 6.8)	32.0 CH_2_	1′
3′	1.62–1.65 (m)	24.1 CH_2_	
(CH_2_)_26_	1.31–1.27 (m)	28.7–28.1 CH_2_	
30	1.44–1.46 (m)	31.9 CH_2_	32′
31′	1.12–1.14 (m)	21.7 CH_2_	32′
32′	0.89 (*t*, *J* = 6.8)	13.1 CH_3_	31′, 30′

### Alkaline hydrolysis of compound 3

Compound **3** (4 mg) was dissolved in five mL of 3% KOH/MeOH solution and kept undisturbed for 15 min at ambient temperature. After 15 min, 1 N HCl/MeOH was added to the solution for the neutralization. The solution was then partitioned with CHCl3, CHCl3 layer was separated, evaporated and the obtained residue was taken up for column chromatography on SiO2 (mesh 60–120) using as eluent hexane: EtOAc gradient to provide methyl ester of dotriacontanoic acid, which was verified by GC/MS (Ibrahim, Mohamed & Ross, 2016; El-Shanawany et al., 2015; Al-Musayeib et al., 2013). A 500-Clarus Perkin-Elmer GC/MS (Waltham, MA, USA) was applied for GC/MS analysis. The integrator combined with software (4.5.0.007 version) controller was turbo mass. A 5 MS/GC elite capillary (30 × 0.25 mm × 0.5 µm) column and helium (He) a carrier gas at 2 ml/min flow rate (55.8 cm/s flow initial with 32 p.s.i., split; 1:40) were applied. Temperature conditions including; source temperature, inlet line temperature, emission trap and electron energy were adjusted at 150 °C, 200 °C, 100 °C and 70 eV, respectively. The injector temperature at 220 °C was maintained. Whereas, the temperature of the column was set at 50 °C for 5 min, raised to 220 °C at the 20 C/min rate. MS was scanned from 50 to 650 m/z.

### Antioxidant activity

### DPPH radical scavenging activity

The ability of the samples to scavenge DPPH radical was determined by Wang, Chen & Hou (2019) method with slight modification (Wang, Chen & Hou, 2019). A total of 20 µL of different sample concentrations (5.25–50 mg mL^−1^) solutions was reacted with 180 µL of DPPH^•^ (6–5 M) dissolved in methanol (80%) in a 96-well plate. Samples were incubated at ambient temperature under dark conditions for 30 min and the absorbance wavelength was measured at 517 nm against a blank sample. DPPH^•^ solution and ascorbic acid were used as blank samples and positive control, respectively. The inhibitory concentration (IC_50_) was expressed as the concentration that inhibits the 50% of DPPH and calculated using the following equation

\begin{eqnarray*}\text{Inhibition percentage}~(\text{%})= \frac{ \left( {\mathrm{A}}_{\mathrm{b}}-{\mathrm{A}}_{\mathrm{s}} \right) }{{\mathrm{A}}_{\mathrm{b}}} \times 100 \end{eqnarray*}

where A_b_ and A_s_ are the absorbance values of the blank and test samples, respectively.

### ABTS radical scavenging activity

ABTS radical cation decolorization assay was performed to measure the total antioxidant activity of the samples by obeying the previously described method (Re et al., 1999). In brief, ABTS^•+^ radicals were generated by treating 7 mM ABTS^+^ aqueous solution with 2.4 mM potassium persulfate for 12-16 h at room temperature in the dark. Prior to use, this solution was diluted with ethanol (approx. 1:89 v/v) and equilibrated to 0. 7,000 ± 0.02 at 300 °C absorbance at 734 nm to obtain the working solution. Afterward, a 1.0 mL of working solution was reacted with 20 µL (1 mg mL^−1^) samples and incubated for 30 min. After incubation, samples were scanned at 734 nm absorbance wavelength and the percentage of inhibition was determined. Ascorbic acid (AA) was applied reference standard.

### Evaluation of cholinesterase inhibitory activities

The cholinesterase inhibitory effect of test sample was investigated on two enzymes (acetylcholinesterase and butyrylcholinesterase) by spectrophotometric method by following Ellman et al. procedure with little modification (Ellman et al., 1961). Crude enzymes, AChE (acetylcholinesterase) and BChE (butyrylcholinesterase) were collected from brain of mice and blood of humans, respectively, by obeying earlier described procedure (Asaduzzaman et al., 2014; Uddin et al., 2015). The AChE and BChE assays were tested by using two chemical substrates acetylthiocholine iodide and S-butyrylthiochoilne iodide, respectively. In brief, 10 µL of each enzyme was reacted individually with equal volume (10 µL) of different concentration (25–400 µgmL^−1^) test sample and reference standard followed by incubation at 37 °C for 15 min for the complete interaction. Afterwards, 2-nitrobenzoic acid (1 mM, 62 µL), sodium phosphate buffer (50 mM, 25 µL, pH 8) provided with bovine albumin serum (0.1%) and acetylcholine iodide (0.5 mm, 13 µL) were added separately into each reaction mixture. Each reaction mixture was individually incubated further for 15 mis at 37 °C and absorbance at 405 nm was immediately noted against the blank. Donepezil and galantamine were used as reference compounds for AChE and BChE activity, respectively. All the experiments were performed in triplicates to avoid error and the results were estimated through the two-tailed Student’s *t*-test at a *p* < 0.05 significance. The inhibition percentage of cholinesterase activity was calculated using the following equation

\begin{eqnarray*}\mathrm{I}\text{%}= \frac{{\mathrm{A}}_{\mathrm{C}}-{\mathrm{A}}_{\mathrm{s}}}{{\mathrm{A}}_{\mathrm{C}}} \times 100 \end{eqnarray*}

where A_c_ and A_s_ is the absorbance of control and sample or reference compound. IC50 values could be determined from the dose response curve obtained by plotting the percent inhibition values against test concentrations of each compound.

### Antimicrobial activity

Minimum inhibitory concentration (MIC) assay was performed to evaluate the *in vitro* antimicrobial activities using a broth microdilution method (Nascente, 2009). Three bacterial strains *Escherichia coli* (ATCC 25922), *Pseudomonas aeruginosa* (ATCC 27853), and *Staphylococcus aureus* were applied to examine the antimicrobial activity of samples. Briefly, the microbial strains were transferred to Muller Hinton agar (MHB, HiMedia, India) and 24-h colonies were individually suspended in 10 mL Muller Hinton broth (MHB, HiMedia, India). The suspension of each microbial strain was standardized at 575 nm wavelength using a spectrophotometer (CRAIC Technologies, CA, USA), to match the McFarland scale (1.5 × 108 CFU mL^−1^). The standardized suspension was further diluted to obtain a final concentration of 5 × 105 CFU mL^−1^. The samples prepared in DMSO at 1 mgmL^−1^ and different concentrations (30–500 µgmL^−1^) were attained after dilution in Mueller Hinton broth. Three inoculated wells containing different concentrations of DMSO (4% to 1% range), one non-inoculated well without an antimicrobial agent and negative controls were included. The inoculated well was used to monitor whether the broth was sufficient for the microbial strain to grow. Chloramphenicol (500 to 30 mgmL^−1^) was applied as a positive control. The sample treated 96-well microplates were sealed and incubated for 24 h at 37 °C After a 1-day incubation, 30 ml of 0.02% resazurin solution was added into each well to examine the viability of the microbial strain (Palomino et al., 2002). The minimum used concentration of test sample that inhibited the microorganism growth (MIC value) was calculated as the minimum concentration of the test samples required to prevent the color change of the resazurin solution. All the assays were performed in triplicates.

### Statistical analysis

The experiments were conducted in triplicates and results were expressed as mean ± standard deviation. Statistical and graphical analysis were performed on Graph Pad Prism (version 8.0.1) and Microsoft Excel 2010. *T*-test was carried out to determine the statistical significance between the average values and (*p* < 0.05) was considered significant.

## Results & Discussion

**Figure 2:**
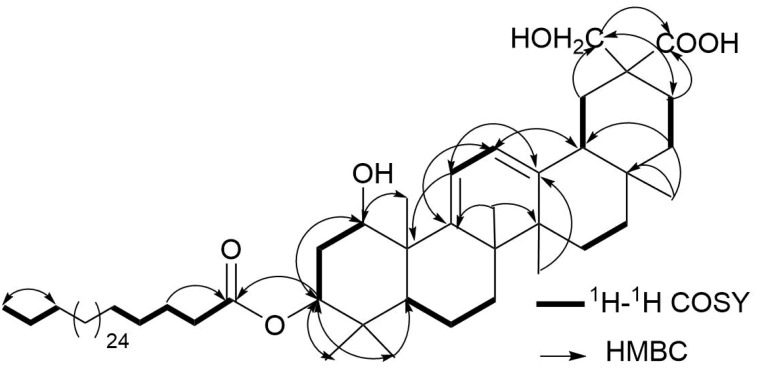
Some Key ^1^H-^1^H COSY (–) and HMBC (H → C) correlations of **3**.

Compound **3** was isolated as a white amorphous powder. It gave a positive Lieberman-Burchard reaction, indicating its triterpenoidal nature (Al-Musayeib et al., 2013; Ibrahim et al., 2012). The molecular formula of **3** was determined as C_62_H_108_O_6_ on the basis of the ESI-MS pseudo-molecular ion peaks at *m/z* 971.4 [M+Na]^+^ and 947.8 [M-H]^−^. The IR spectrum showed the presence of hydroxyl (3,420 cm^−1^), ester carbonyl (1,732 cm^−1^), acid carbonyl (1,712 cm^−1^), and double bond (1,636 cm^−1^) functionalities. The ^13^C and DEPT NMR spectra of **3** displayed resonances for 62 carbons: six tertiary methyl, one primary methyl, 39 methylenes, one of them for an oxymethylene δ(C) 69.6 (C-30), six methines, and ten quaternary carbon signals, including one ester carbonyl at δ(C) 172.3 (C-1′), acid carbonyl δ(C) 173.0 (C-29), and two quaternary olefinic carbons at δ(C) 149.5 (C-9) and 146.0 (C-13). The ^1^H NMR spectrum showed six methyl singlets at δ(H) 0.97 H-C(23), 0.91 H-C(24), 1.26 H-C(25), 1.17 H-C(26), 1.07 H-C(27), and 0.92 H-C(28), characteristic for an oleanane-type triterpenoid (Ibrahim, Mohamed & Ross, 2016; Al Musayeib et al., 2014; Mahato & Kundu, 1994). They correlated to the carbon signals, resonating at δ(C) 24.6, 15.8, 22.5, 19.4, 19.2, and 27.2, respectively in the HSQC spectrum. Moreover, two coupled olefinic protons at δ(H) 5.73 (*d*, *J* = 6.0, H-C(11)) and 5.58 (*d*, *J* = 6.0, H-C(12)) were observed in the ^1^H NMR and 1H-1H COSY spectra, indicating the presence of two tri-substituted olefinic double bonds (Fig. 2). They showed HSQC cross peaks to the carbons at δ(C) 116.0 C(11) and 119.6 C(12). The placement of the double bonds at C9-C11 and C12-C13 was established based on the HMBC cross peaks of H-C(11) to C(8), C(9), C(10), and C(13), H-C(12) to C(9), C(13), and C(14), H-C(18) to C(12) and C(13), H-C(27) to C(13), and H-C(25) and H-C(26) to C(9), indicating that **3** had an olean-9(11),12-diene skeleton (Caceres-Castillo et al., 2008; Mahato & Kundu, 1994) (Fig. 2). The ^1^H and ^13^C NMR spectra displayed two oxymethine signals at δ(H) 4.17 (*d*, *J* = 2.8, H-C(1))/δ(C) 71.6 (C-1) and 5.09 (dd, *J* = 12.0, 5.0 Hz, H-C(3))/74.3 (C-3). Their assignment was established based on the observed 1H-1H COSY of H-C(2) to H-C(1) and H-C(3) as well as the HMBC cross peaks of H-C(1), H-C(23), and H-C(24) to C(3) and H-C(3) and H-C(25) to C(1). The coupling constants of H-C(1) (*J*_1,2_ = 2.8 Hz) and H-C(3) (*J*_3,2_ = 12.0, 5.0 Hz) revealed that the hydroxyl groups at C(1) and C(3) were α- and β-configured, respectively, based on comparison with the previously reported triterpenoids [*J*_1,2_ = 2.5–3.0 Hz and *J*_3,2_ = 10.0–12.8, 4.2–7.2 Hz] (Ibrahim et al., 2019; Litaudon et al., 2009; Rogers & Subramony, 1988). The signals at δ(H) 4.06 and 3.81 (2H, each *d*, *J* = 10.2, H-C(30))/ δ(C) 69.6 (C-30) and δ(C) 173.0 (C-29) revealed the presence of an oxymethylene and carboxyl functionalities in **3**. Their placement at (C-20) was established by the HMBC cross peaks of H-C(19) and H-C(21) to (C-29) and (C-30) and H-C(30) to (C-29). The multiple aliphatic protons at δ(H) 1.31–1.27 were attributed to the presence of a long chain aliphatic moiety. The ^13^C NMR signal at δ(C) 172.3 (C-1′) was assigned to an ester carbonyl, which was confirmed by the IR absorption band at 1,732 cm^−1^. While the signals at δ(H) 0.89 (t, *J* = 6.8)/ δ(C) 13.1 were assigned to the terminal methyl group of a straight-chain fatty acid (Al-Musayeib et al., 2013; El-Shanawany et al., 2015; Ibrahim, Mohamed & Ross, 2016). Upon alkaline hydrolysis of 3, it gave a methyl ester of dotriacontanoic acid, which was identified by GC-MS molecular ion peak at *m/z* 494 [M]^+^ andconfirmed by the ESI-MS fragment ion peak at *m/z* 486 [M+H-(dotriacontanoyl moiety)]^+^. The attachment of the fatty acyl moiety at (C-3) was established by the HMBC cross peak of H-C(3) to (C-1′) and confirmed by the downfield shift of HC-(3) δ(H) 5.09. The relative stereochemistry at stereocenters was assigned by comparing the *J* values and ^1^H and ^13^C chemical shifts with those of related triterpenes (Ibrahim et al., 2019; Litaudon et al., 2009). Based on these findings, **3** was assigned as 1 α,3 β,30-trihydroxy-29-carboxy-olean-9(11),12-diene-3-dotriacontanoate and named abubidentin A.

The known compounds were identified as 2-hydroxydocosanoic acid (**1**) (Inagaki et al., 2001) and stigmasta-22-ene-3-β-ol (**2**) (Connor et al., 2006) by comparing their NMR spectral and physical data in the literature.

### Antioxidant activity assessments

**Figure 3:**
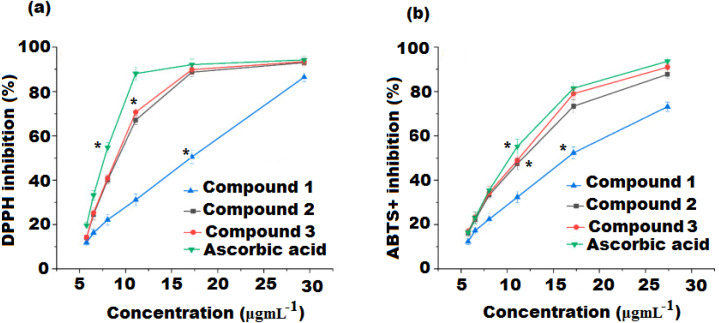
(A) DPPH and (B) ABTS free radical scavenging activity of compound **1**, **2**, *3* and ascorbic acid. Values were obtained as mean ± standard deviation and *mean significant difference compared to control (*p* < 0.05).

DPPH and ABTS^+^ assays are widely applied to measure the compound’s ability to determine its antioxidant potential. Both the spectrophotometric methods used for evaluating antioxidant activity are based on electron transfer reactions and visually rely on the reduction of a colored oxidant. The obtained results from these two assays expressed good correlation. Fig. 3A and Table 2 represents the free radical inhibition of isolated compounds (**1**, **2**, and **3**) and ascorbic acid (AA, standard) at different concentrations. The results showed that the DPPH scavenging potential of compounds **1**, **2**, and **3** incrementally increased with the increase in concentration of compounds. The IC_50_ values obtained were 10.82 ± 0.24, 7.60 ± 0.42, and 4.67 ± 0.28 µgmL^−1^ for compounds **1**, **2**, and **3** were, respectively, indicating that compound **3** possesses the highest radical scavenging potential and was 1.55 fold lower than ascorbic acid (IC_50_ 3.12 ± 0.24 µgmL^−1^), followed by compounds **2** and **1** (*p* < 0.05).

**Table 2:** Antioxidant activity and cholinesterase inhibitory of the extract and pure isolated compounds (**1**, **2** and **3**) from of *A bidentatum*.

Sample	Antioxidant activity		Cholinesterase inhibitory	
	DPPH IC_50_ (µgmL^−1^)	ABTS^+^ IC_50_ (µgmL^−1^)	AChE IC_50_ (µgmL^−1^)	BChE IC_50_ (µgmL^−1^)
Ethanol extract	16.34 ± 0.25e	18.65 ± 0.67e	132.56 ± 1.4e	142.35 ± 0.54e
Compound **1**	10.82 ± 0.24^d^	11.45 ± 0.37^d^	121.97 ± 1.61^d^	137.76 ± 0.67^d^
Compound **2**	7.60 ± 0.42^c^	8.18 ± 0.13^c^	68.65 ± 0.56^c^	49.52 ± 0.35^c^
Compound **3**	4.67 ± 0.28^b^	6.42 ± 0.25^b^	38.13 ± 0.07^b^	32.68 ± 0.37^b^
Ascorbic acid	3.12 ± 0.24^a^	4.45 ± 0.17^a^	–	–
Donepezil	–	–	9.32 ± 0.38^a^	–
Galantamine	–	–	–	10.27 ± 0.88^a^

**Notes.**

Means in each column with different subscript letters (a, b, c, d, e) differ significantly (*P* < 0.05).

The ABTS^+^ assay is an additional important procedure for the quantification of radical scavenging potential that can provide parallel results to those obtained in the DPPH assay. The results showed that all the three isolated compounds exerted significant ABTS free radical scavenging activity and had an antioxidant potential proportional to that of ascorbic acid (Fig. 3B and Table 2). Compound **3** was found to be the most active radical scavenger with an IC_50_ value of 6.42 ± 0.25 µgmL^−1^ and 3.12 fold lower than that of ascorbic acid, followed by compound 2 and 1 with IC_50_ values of 8.18 ± 0.13 and 11.45 ± 0.37 µgmL^−1^, respectively ( *p* < 0.05). Compound **3** displayed both the highest DPPH and ABTS radical scavenging activities. A correlation between DPPH and ABTS methods applied to determine the antioxidant potential of the tested compounds was examined. The DPPH radical activity showed a strong correlation with ABTS radical activity (R_2_ = 96.67%).

### Cholinesterase inhibitory activity

**Figure 4:**
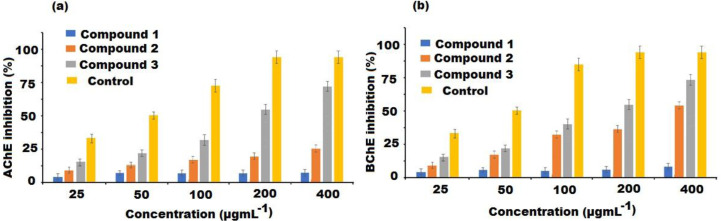
Cholinesterase inhibitory activities of compound **1–3** isolated from *A. bidentatum* (a) Inhibition of acetylcholinesterase (AChE) by isolated compounds of *A. bidentatum* and standard donepezil, (B) Inhibition of butyrylcholinesterase (BChE) by compounds isolated from *A. bidentatum* and standard galantamine.

The soluble fraction of the extract and compounds (**1–3**) were screened for AChE and BChE inhibition at different concentrations. The percent inhibition of AChE by the test compounds were presented in Fig. 4A. Donepezil used as a reference AChE inhibitor showed an IC_50_ value of 9.32 ± 0.38 µgmL^−1^. The result revealed that all the investigated compounds (**1–3**) showed inhibition of the AChE enzyme in a dose-dependent manner. Among the compounds, high activity was displayed by compound **3** with IC_50_ values of 38.13 ± 0.07 µgmL^−1^, followed by compound **2** (IC_50_ value = 68.65 ± 0.56 µgmL^−1^). However, compound **1** showed low activity with IC_50_ of 121.97 ± 1.61 µgmL^−1^ (Table 2). Similarly, in BChE inhibitory assay, compounds **3** and **2** exerted high inhibitory potentials with IC_50_ of 32.68 ± 0.37 and 49.52 ± 0.35 µgmL^−1^, respectively (Table 2, Fig. 4B). The IC_50_ value of compound **1** was 137.76 ± 0.67 µgmL^−1^ exhibited very negligible effects. Thus, compound **3** had appreciable activity towards both AChE and BChE enzymes.

### Antimicrobial activity

The antimicrobial potential of isolated compounds (**1**–**3**) was assessed by calculating MIC values and their ability to inhibit the growth of the tested microbial strain. The assay was performed in 96-well microplates by applying resazurin as a developer. The well plates that exhibited the blue color after the addition of resazurin were considered as the MIC value for each microorganism (Ostrosky et al., 2008). The pure isolated compounds **1–3** were examined against *E. coli*, *P. aeruginosa,* and *S. aureus* bacterial strains for their antimicrobial potential. All the tested compounds exerted antimicrobial effect in the range of 125–1,000 µgmL^−1^ towards the pathogenic strains (Table 3). However, the prominent activity was shown by compound 2 with MIC ≤125 gmL^−1^, ≤250 µgmL^−1^, ≤150 µgmL^−1^ and compound 3 with ≤150 µgmL^−1^, ≤125 µgmL^−1^, ≤125 µgmL^−1^, against *E. coli, P. aeruginosa*, and *S.aureus*, respectively. There is no consistent classification with respect to MIC values (Aligiannis et al., 2001), but the values obtained ≤1,000 µgmL^−1^ were considered as satisfactory and sensitive (Webster et al., 2008). Thus, the MIC value of compounds **3** and **2** can be considered as promising.

**Table 3:** Minimum inhibitory concentration (µgmL^−1^) of extract and pure isolated compounds (**1, 2** and **3**) against selected pathogens.

**Sample**	**Microbial strain**		
	***E. coli***	***P. aeruginosa***	***S. aureus***
Ethanol extract	≤600	≤1,000	≤1,000
Compound **1**	≤500	≤1,000	≤1,000
Compound **2**	≤125	≤250	≤150
Compound **3**	≤150	≤125	≤125
Chloramphenicol	≤40	≤40	≤40

## Conclusion

A new oleanane-type triterpene ester, namely abubidentin A together with two known 2-hydroxydocosanoic acid and stigmasta-22-ene-3-*β*-ol were isolated from aerial parts of *A.bidentatum*. The extracts and compounds were investigated for antioxidant, cholinesterase inhibitory, and antimicrobial activities. The outcomes demonstrated that the newly isolated compound possesses a strong antioxidant effect towards DPPH and ABTS+ radical scavenging assays. This new triterpene exhibited high inhibition against acetylcholinesterase and butyrylcholinesterase. In addition, the new compound also showed promising antimicrobial effects against tested microorganisms. These results suggested that *A. bidentatum* is a promising source of useful natural products and the new compound offers opportunities to develop a novel drug.

## Supplemental Information

10.7717/peerj.13040/supp-1 Supplemental Information 1 Supplemental Figures and Tables

10.7717/peerj.13040/supp-2 Supplemental Information 2 Raw data

## Additional Information and Declarations

### Data Availability

The following information was supplied regarding data availability: The raw data is available in the Supplemental File.
