# Supplementary material for: Abubidentin A, New Oleanane-type Triterpene Ester from Abutilon bidentatum and its antioxidant, cholinesterase and antimicrobial activities
Source: PeerJ. 2022 Mar 8;10:e13040. doi: 10.7717/peerj.13040 (PMC8916034; doi:10.7717/peerj.13040)
Supplement: Supplemental Information 1 [file peerj-10-13040-s001.docx]

**Supplementary Information**

**Abubidentin A, New Oleanane-type Triterpene Ester from *Abutilon bidentatum*** **and its antioxidant, cholinesterase and antimicrobial activities**

Gadah A. Al-Hamoud^1*^, Nawal M. Al Musayeib^1*^, Musarat Amina^1^, Sabrin R. M. Ibrahim^2^

^1^Department of Pharmacognosy, College of Pharmacy, King Saud University, Riyadh 11451, Saudi Arabia.

^2^Department of Pharmacognosy, Faculty of Pharmacy, Assiut University, Assiut 71526, Egypt.

*Corresponding author: Nawal M. Al Musayeib [nalmusayeib@ksu.edu.sa](mailto:nalmusayeib@ksu.edu.sa)

Gadah A. Al-Hamoud [gadhamoud@ksu.edu.sa](mailto:gadhamoud@ksu.edu.sa)

**List of supplementary materials**

| **Figure S1.** ^1^H NMR spectrum of compound **3** (500 MHz, CDCl_3_). |
| --- |
| **Figure S2.** ^13^C NMR spectrum of compound **3** (176 MHz, CDCl_3_). |
| **Figure S3.** DEPT ^13^C NMR spectrum of compound **3** (176 MHz, CDCl_3_). |
| **Figure S4.** ^1^H-^1^H COSY spectrum of compound **3**. |
| **Figure S5.** HSQC spectrum of compound **3**. |
| **Figure S6.** HMBC spectrum of compound **3.** |
| **Figure S7.** Negative ESIMS spectrum of compound **3**. |
| **Figure S8.** Positive ESIMS spectrum of compound **3**. |
| Table S1. Determination of DPPH activity of isolated compound **1**, **2**, and **3** from of *A. bidentatum* |
| Table S2. Determination of ABTS+ activity of isolated compound **1**, **2** and **3** from of *A. bidentatum* |
| Table S3. Determination of AChE inhibitory activity of isolated compound **1**, **2** and **3** from of *A. bidentatum* |


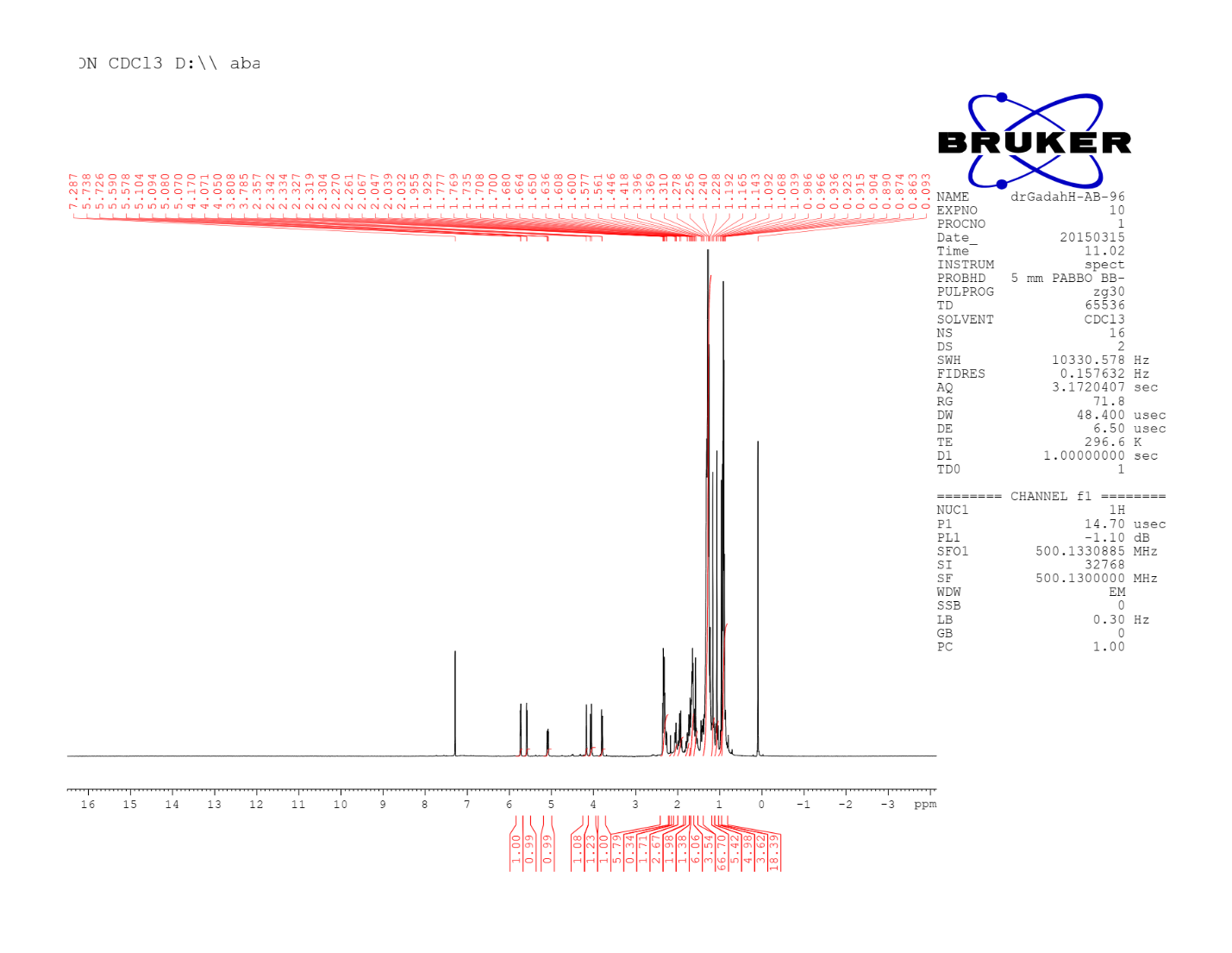


**Figure S1.** ^1^H NMR spectrum of compound **3** (500 MHz, CDCl_3_).


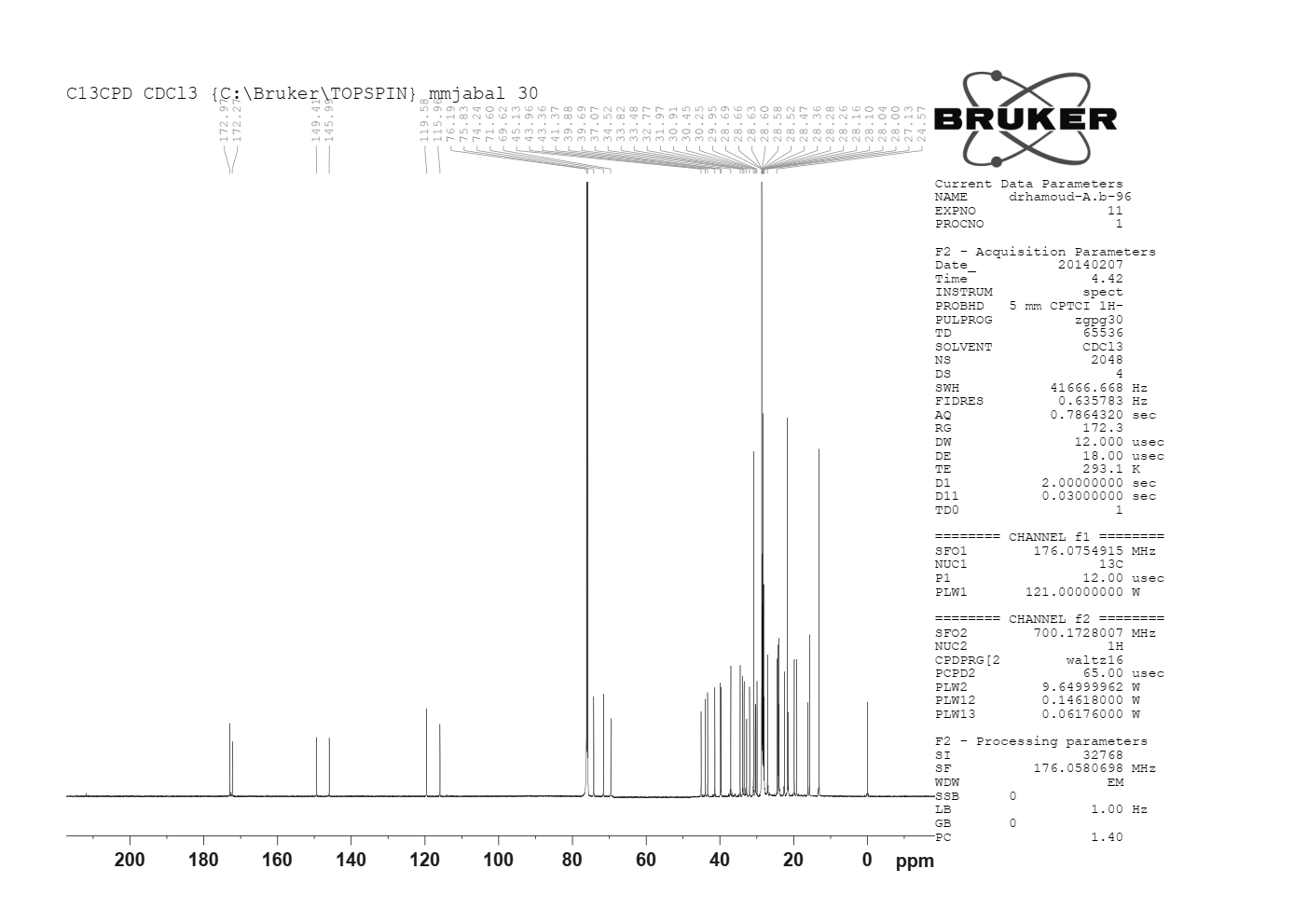


**Figure S2.** ^13^C NMR spectrum of compound **3** (176 MHz, CDCl_3_).


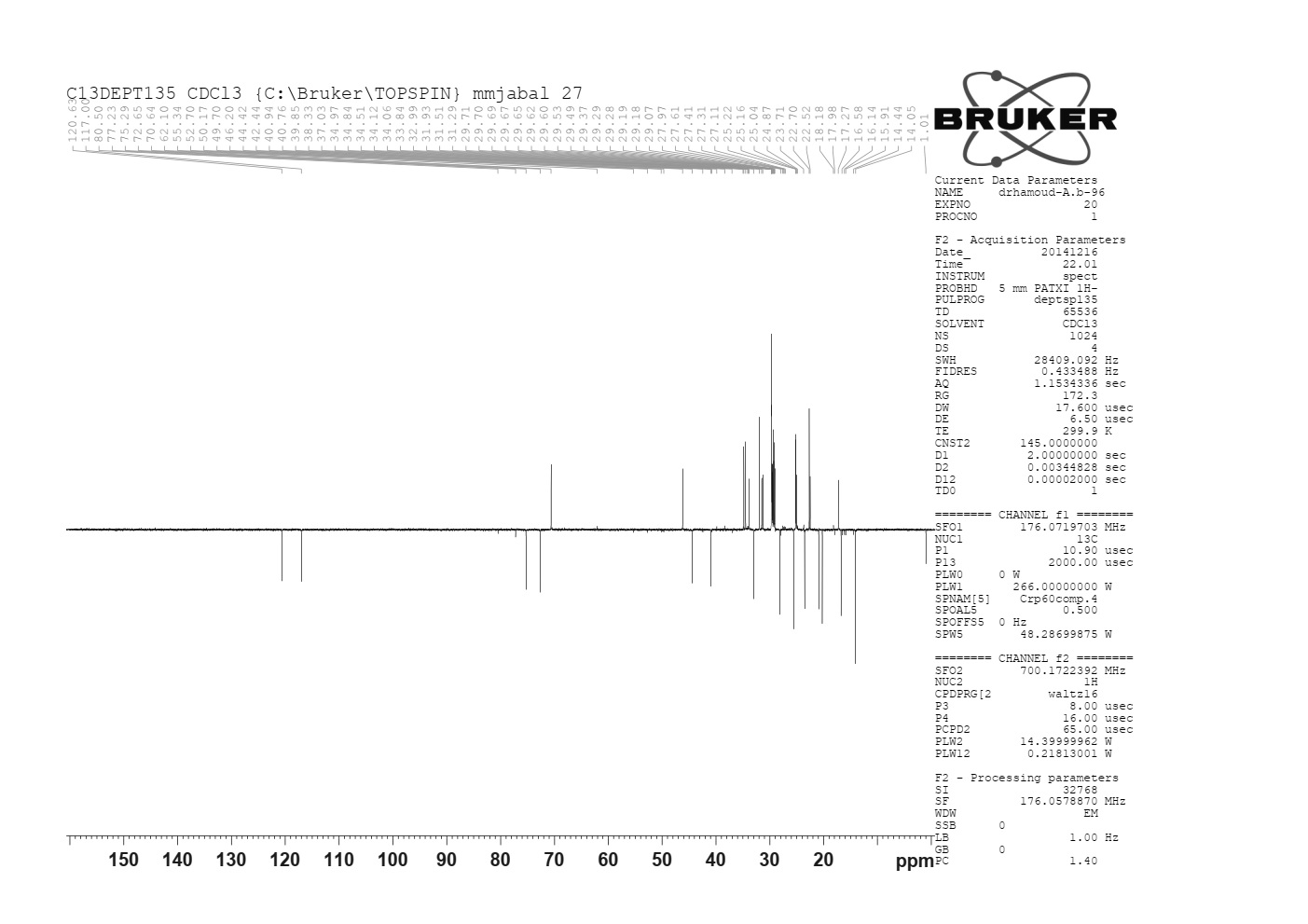


**Figure S3.** DEPT ^13^C NMR spectrum of compound **3** (176 MHz, CDCl_3_).


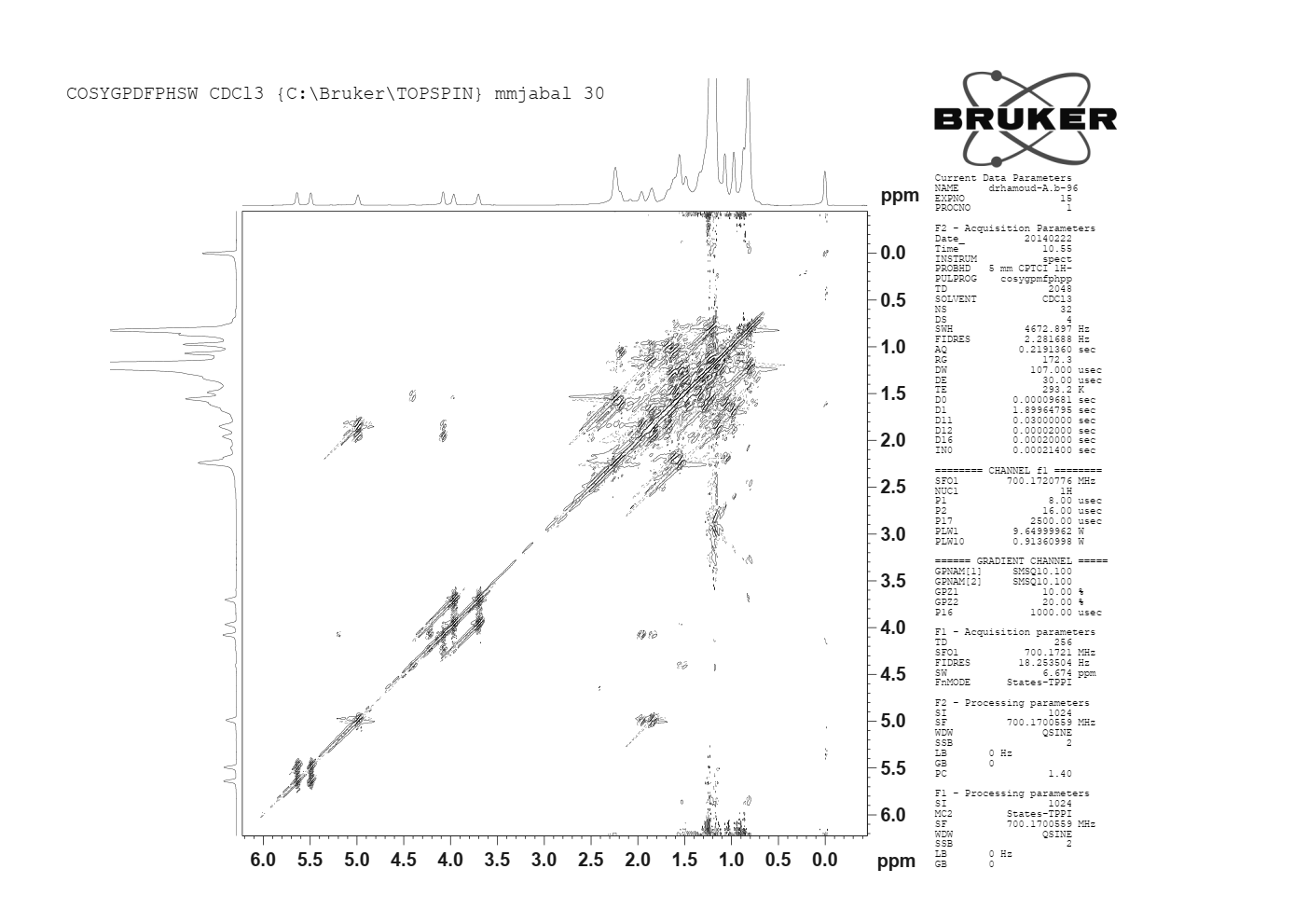


**Figure S4.** ^1^H-^1^H COSY spectrum of compound **3**.


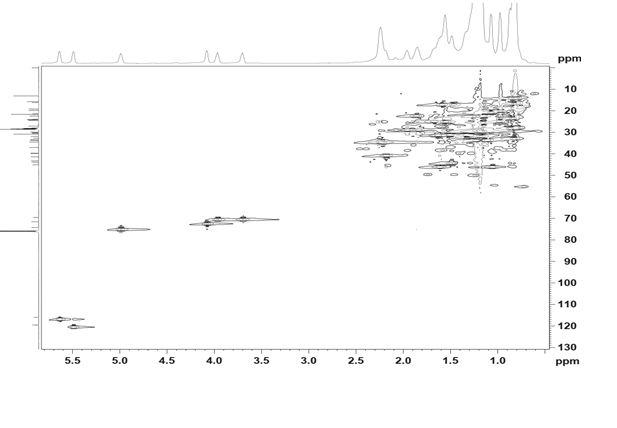


**Figure S5.** HSQC spectrum of compound **3**.


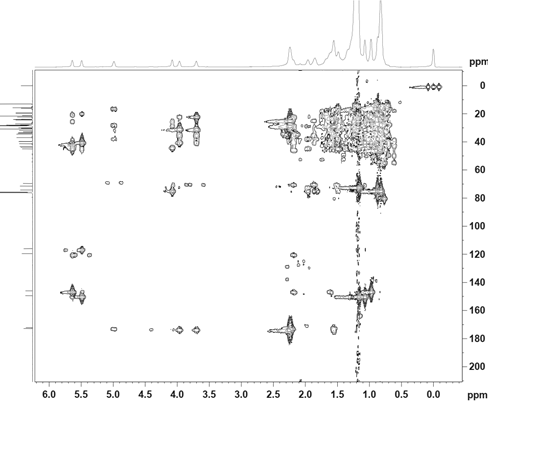


**Figure S6.** HMBC spectrum of compound **3.**


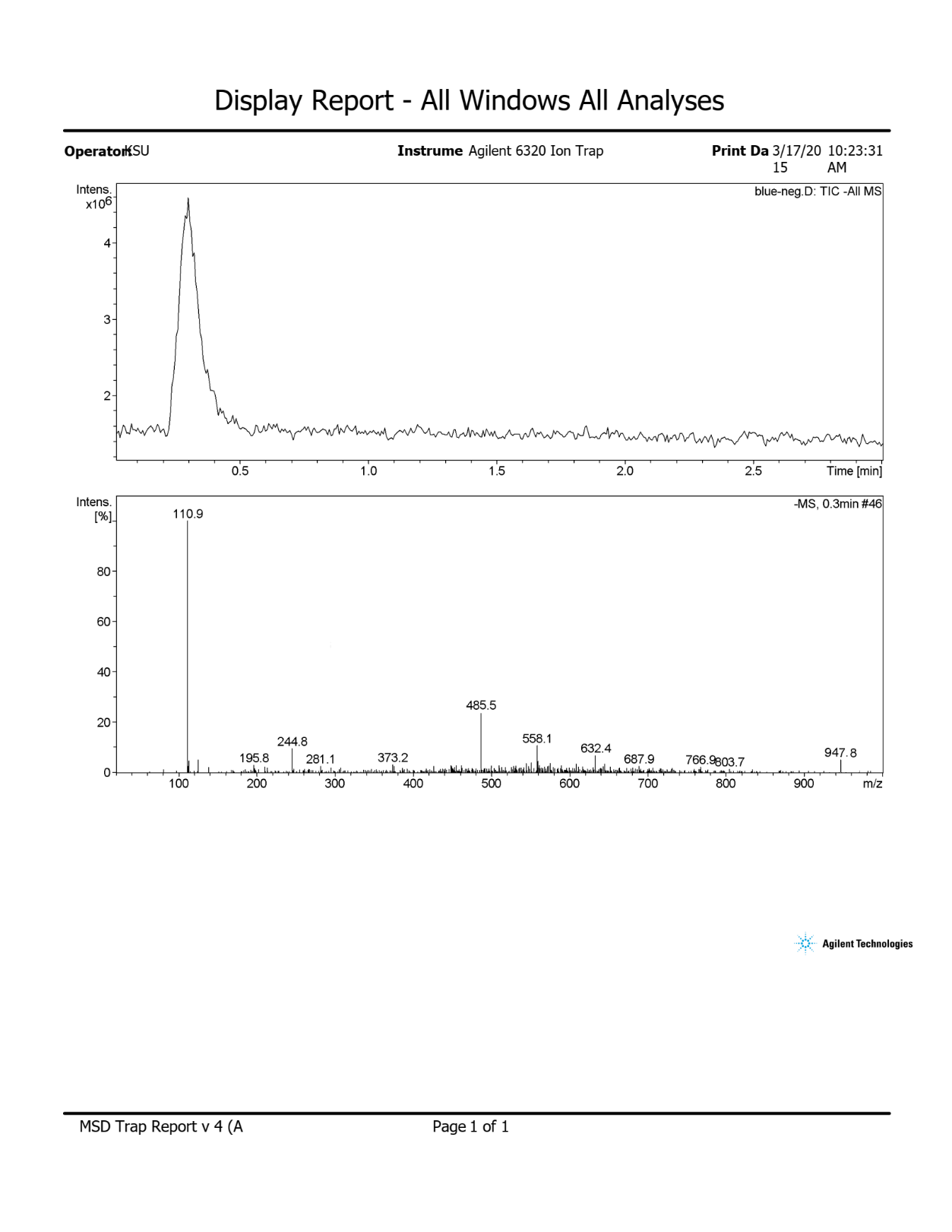


**Figure S7.** Negative ESIMS spectrum of compound **3**.


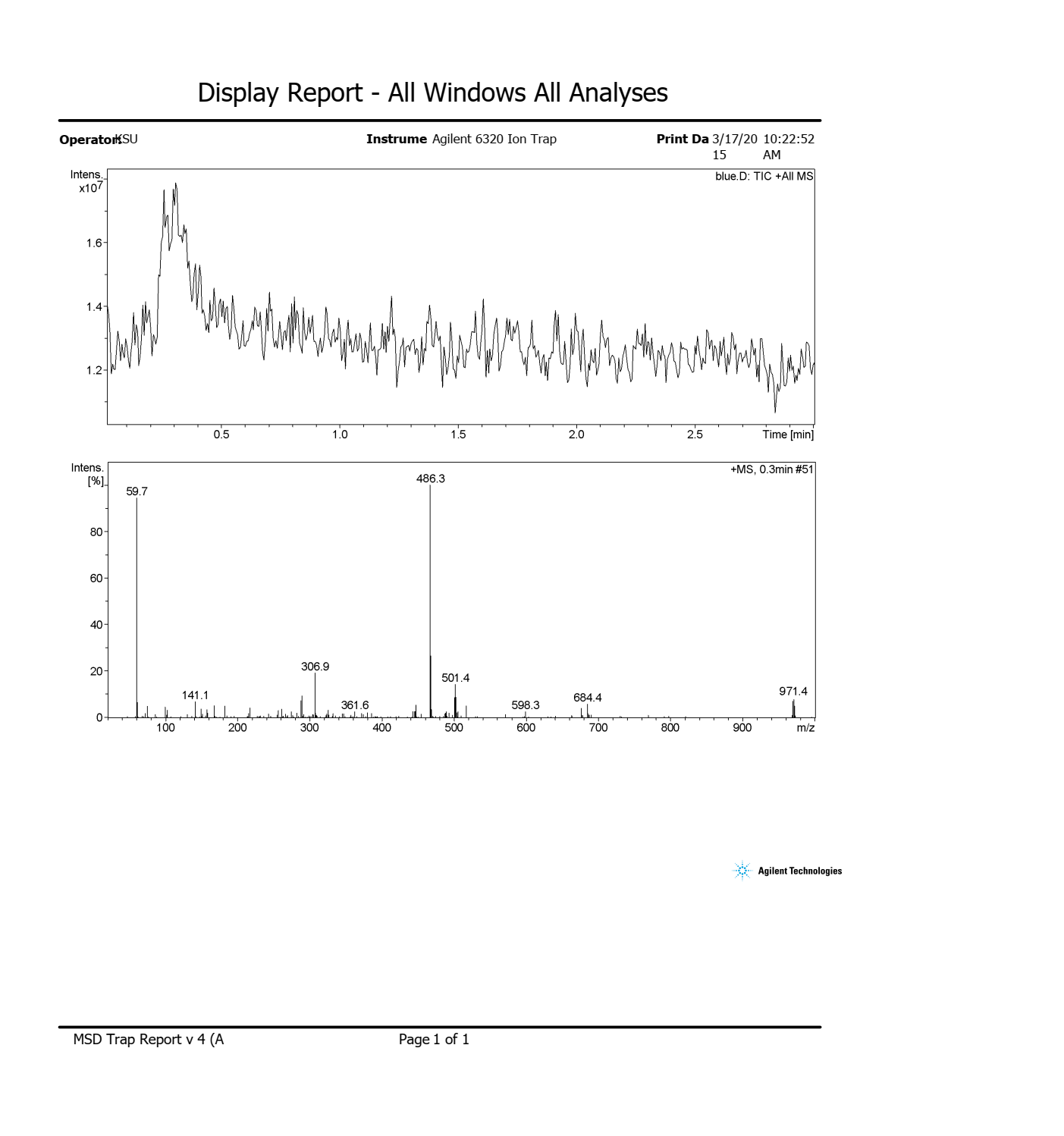


**Figure S8.** Positive ESIMS spectrum of compound **3**.

Table S1. Determination of DPPH activity of isolated compound **1**, **2**, and **3** from of *A. bidentatum*

| **Sample** | **Conc (µg/mL)** | **No. of trials** | **Abs** | **AA (mg/gm)** | | |
| --- | --- | --- | --- | --- | --- | --- |
|  |  |  |  |  | **Avg** | **SD** |
| **1** | 20 | A | 0.474 | 10.98 |  |  |
|  | 20 | B | 0.468 | 10.54 | **10.82** | **0.24** |
|  | 20 | C | 0.476 | 10.96 |  |  |
| **2** | 20 | A | 0.454 | 7.84 |  |  |
|  | 20 | B | 0.458 | 7.12 | **7.60** | **0.42** |
|  | 20 | C | 0.460 | 7.86 |  |  |
| **3** | 20 | A | 0.515 | 4.89 |  |  |
|  | 20 | B | 0.512 | 4.35 | **4.67** | **0.28** |
|  | 20 | C | 0.489 | 4.78 |  |  |
| **Ascorbic acid** | 20 | A | 0.515 | 3.30 |  |  |
|  | 20 | B | 0.513 | 2.84 | **3.12** | **0.24** |
|  | 20 | C | 0.517 | 3.22 |  |  |

Table S2. Determination of ABTS+ activity of isolated compound **1**, **2** and **3** from of *A bidentatum.*

| **Sample** | **Conc. (µg/mL)** | **No. of trials** | **Abs** | **AA (mg/gm)** | | |
| --- | --- | --- | --- | --- | --- | --- |
|  |  |  |  |  | **Avg** | **SD** |
| **1** | 20 | A | 0.685 | 11.64 |  |  |
|  | 20 | B | 0.680 | 11.02 | **11.45** | **0.37** |
|  | 20 | C | 0.670 | 11.70 |  |  |
| **2** | 20 | A | 0.578 | 8.25 |  |  |
|  | 20 | B | 0.572 | 8.02 | **8.18** | **0.13** |
|  | 20 | C | 0.574 | 8.28 |  |  |
| **3** | 20 | A | 0.515 | 6.56 |  |  |
|  | 20 | B | 0.512 | 6.13 | **6.42** | **0.25** |
|  | 20 | C | 0.489 | 6.58 |  |  |
| **Ascorbic acid** | 20 | A | 0.512 | 4.52 |  |  |
|  | 20 | B | 0.515 | 4.25 | **4.45** | **0.17** |
|  | 20 | C | 0.518 | 4.58 |  |  |

Table S3. Determination of AChE inhibitory activity of isolated compound **1**, **2** and **3** from of *A bidentatum.*

| **Sample** | **Conc (µg/mL)** | **No. of trials** | **Abs** | **Donep (mg/gm)** | | |
| --- | --- | --- | --- | --- | --- | --- |
|  |  |  |  |  | **Avg** | **SD** |
| **1** | 10 | A | 0.342 | 122.89 |  |  |
|  | 10 | B | 0.328 | 120.10 | **121.97** | **1.61** |
|  | 10 | C | 0.345 | 122.92 |  |  |
| **2** | 10 | A | 0.312 | 68.94 |  |  |
|  | 10 | B | 0.315 | 68.01 | **68.65** | **0.56** |
|  | 10 | C | 0.317 | 69.02 |  |  |
| **3** | 10 | A | 0.289 | 38.16 |  |  |
|  | 10 | B | 0.294 | 38.05 | **38.13** | **0.07** |
|  | 10 | C | 0.315 | 38.20 |  |  |
| **Donepezil** | 10 | A | 0.256 | 9.52 |  |  |
|  | 10 | B | 0.262 | 8.87 | **9.32** | **0.38** |
|  | 10 | C | 0.264 | 9.58 |  |  |

Table S4. Determination of BChE inhibitory activity of isolated compound **1**, **2** and **3** from of *A bidentatum.*

| **Sample** | **Conc (µg/mL)** | **No. of trials** | **Abs** | **Gal (mg/gm)** | | |
| --- | --- | --- | --- | --- | --- | --- |
|  |  |  |  |  | **Avg** | **SD** |
| **1** | 10 | A | 0.375 | 138.12 |  |  |
|  | 10 | B | 0.379 | 136.98 | **137.76** | **0.67** |
|  | 10 | C | 0.354 | 138.18 |  |  |
| **2** | 10 | A | 0.355 | 49.70 |  |  |
|  | 10 | B | 0.365 | 49.12 | **49.52** | **0.35** |
|  | 10 | C | 0.358 | 49.74 |  |  |
| **3** | 10 | A | 0.285 | 32.88 |  |  |
|  | 10 | B | 0.286 | 32.25 | **32.68** | **0.37** |
|  | 10 | C | 0.287 | 32.92 |  |  |
| **Galantamine** | 10 | A | 0.345 | 10.78 |  |  |
|  | 10 | B | 0.348 | 9.29 | **10.27** | **0.88** |
|  | 10 | C | 0.352 | 10.80 |  |  |
